# Beyond shelter: a scoping review of evidence on housing in resettlement countries and refugee mental health and wellbeing

**DOI:** 10.1007/s00127-025-02851-1

**Published:** 2025-03-05

**Authors:** Sheenagh McShane, Karen Block, Emma Baker, Yuxi Li, Rebecca Bentley

**Affiliations:** 1 Melbourne School of Population and Global Health, University of Melbourne, Melbourne, Australia; 2 Australian Centre for Housing Research, University of Adelaide, Adelaide, Australia

**Keywords:** Refugees, Asylum seekers, Housing, Resettlement, Mental health, Wellbeing

## Abstract

**Purpose:**

The number of displaced people globally has rapidly increased in the past decade. Housing is an important social determinant of health and a key contributor to poor health outcomes in refugee and asylum seeker populations. It is important to examine evidence for how housing impacts the mental health of refugees and asylum seekers. This review seeks to analyse the research describing how housing conditions and policies are associated with refugee mental health and wellbeing in high-income resettlement countries (such as the United States, Canada, and Australia).

**Methods:**

A scoping review identified forty-four relevant studies. These studies examined various aspects of housing and/or accommodation and their association with mental health and wellbeing in resettled refugee populations.

**Results:**

We found evidence of a relationship between four domains of housing—policy, suitability, environment and time—and mental health. Furthermore, we found evidence that refugees settling in high income countries experienced significant housing issues. Overall, problems with housing quality, location, accessibility (i.e., the nature of systems that govern access to housing) and suitability were associated with poorer mental health outcomes.

**Conclusions:**

In high-income countries, the lack of choice and agency regarding housing contributed to poor mental health outcomes among refugees and asylum seekers. Policies and practices should prioritise the quality, suitability, and accessibility of refugee housing, look at ways to increase choice and agency in resettlement.

## Introduction

Globally the number of people displaced due to political instability, war, conflict and persecution has more than doubled over the last decade from 43 to 108.4 million [1]. Of concern, 2022 saw the 16th year of annual increases in the number of people displaced due to conflict. Most displaced people (62.5 million) remained in their country of origin while about one-third are refugees or asylum seekers^1^ in other countries. The remaining are displaced Venezuelans residing in neighbouring Latin American countries [1]. Although only a small proportion of the world’s refugees are resettled in high-income countries, high rates of mental illness have been reported amongst humanitarian migrants compared to other migrant groups and the general population of the host country [2–6]. Research suggests that trauma exposure and post-migration socioeconomic hardship continue to negatively affect the mental health of refugee populations across generations [3]. Further, there is evidence that post-migration factors have a greater impact on mental health when refugees have also experienced traumatic pre-migration events [7]. Post-migration stressors experienced by refugees such as discrimination [8, 9], social exclusion [8, 10], language barriers [8, 11–13] and housing issues [11, 14, 15] have been associated with both contemporaneous and future mental ill health. Although pre-migration trauma is a predictor for mental disorders, the post-migration context is a significant determinant of mental health and should thus be considered as an appropriate point for therapeutic and psychosocial interventions [16].

Housing is an important social determinant of health and has been identified as a key contributor to poor health outcomes in refugee and asylum seeker populations [17]. Accessing affordable and suitable housing is a challenge for all low-income earners, however refugees face a range of additional barriers, which hinders their ability to find and maintain sustainable housing including pre-migration trauma, discrimination and lack of social capital in their new country [10, 15]. Housing provides not only shelter but security, a base to establish connections and a sense of belonging in a new life [8, 13, 18]. Despite varied policy responses across jurisdictions, the provision of suitable housing is still largely unmet. While there has been research on the importance of housing for protecting the mental health of refugees, this evidence has not been systematically scoped to date.

Previous research suggests that secure housing plays a crucial role in the wellbeing of newly arrived migrants. When migrants have stable accommodation, they are better positioned to engage with community, develop a sense of safety and rebuild their lives [19–22]. These factors contribute significantly to overall wellbeing and successful integration. Conversely, residential instability has been linked to weakened social connections, poorer mental health, and reduced financial resources[15, 23–25]. Asylum seekers face unique hardships in addition to those experienced by resettled refugees. Long periods in the asylum process, being in limbo and fear of deportation, separation from family, and financial instability contribute to poor mental health. Asylum seekers face additional restrictions in access to housing, healthcare, employment, education and financial support which contribute significantly to negative mental health consequences [4, 26–28].

This paper aims to synthesise available evidence regarding the following research questions: This should be on a new line1. What is the association between housing conditions and the mental health and wellbeing of refugees?

2. Which key housing factors impact refugee mental health, and at what points in the settlement journey do these factors provide the greatest benefits?

These research questions build upon the previous work by Ziersch et al. (2018), which showed that factors such as affordability, suitability, tenure, and mobility negatively affected the health of refugees, while physical aspects of housing were linked to poor health [15]. The authors are expanding on this foundation by specifically focusing on mental health and wellbeing, as well as examining the timing of access and support for housing and when they provide the greatest benefit.

## Methods

A scoping review methodology was chosen to systematically analyse the nature and extent of existing peer-reviewed and grey literature, identify research gaps, and comprehensively synthesise the available evidence [29]. This approach is useful when the topic has not been extensively previously reviewed—as is the case for this review [30]. The review adhered to PRISMA guidelines [31, 32]. Arksey and O’Malley’s scoping review methodology guided the review as follows: (1) we identified the research question, (2) we identified relevant studies, (3) we selected relevant studies, (4) we charted the data and (5) we collated, summarised and reported results [31]. This well-accepted framework provides a consistent and rigorous approach for scoping reviews.

### Search strategy

An iterative search strategy was applied that included searching the literature, refining search terms, and reviewing the literature. Electronic databases (Medline, Web of Science, PsychInfo, Embase and socINDEX) were targeted as top research databases for health research. Grey literature searches were also conducted using Google (searching site:.edu or. gov and filetype:.pdf). Study identification included both electronic and manual searching strategies. Electronic searches involved the electronic databases and search terms listed. Additionally, reference lists of articles that were included in a full-text review were hand-searched for additional relevant articles.

The Population Intervention Comparison Outcome (PICO) tool was used to frame the search term concepts for our research question. Comparison was not included as we not comparing with other housing groups. The characteristics of the population, the intervention, and the outcome were defined as follows: population: refugees, asylum seekers, and forced migrants displaced to another country. Other categories of migrants such as labour migrants, spousal or family reunion migrants were excluded as their legal entitlements and migration pathways are different. Intervention: housing was broadly defined to include any aspect of housing or accommodation including its physical nature, quality, access, mobility, security, over-crowding, and location. Outcome: mental health and wellbeing were defined following the multi-dimensional definition provided by the World Health Organization [33], and encompassing mental and wellbeing domains. See Appendix A for the search terms used.

Inclusion Criteria: all studies which reported on the relationship between mental health and wellbeing and housing for refugees and asylum seekers in high-income countries (using the World Bank definition) were included. All study designs were included, encompassing qualitative, quantitative, and mixed methods designs. Grey literature was included to capture relevant research published as reports. Exclusion criteria: studies of migrants, displaced persons, conducted in refugee camps, in low- or middle-income countries were excluded. Dissertations, books, letters to the editor, conference proceedings, reviews or opinion articles were excluded.

### Mental health and wellbeing outcomes

Mental health (MH) was conceptualised and measured across four domains: psychiatric disorders, psychological distress, psychological wellbeing and psychosocial wellbeing. Different studies focused on different domains, each provides a distinct perspective on mental health, and understanding all four provides a more comprehensive understanding of an individual’s mental health status. Psychiatric disorders are a diagnostic category of mental health characterised by clinically significant disturbances in an individual’s cognition, emotional regulation, or behaviour [34]. Psychological distress is characterised by non-specific negative emotional responses to stimuli and often embedded within the context of strain, stress, and distress [35]. Psychological wellbeing refers to positive emotional experiences, such as feeling happy, content and a sense of satisfaction with life. Psychological wellbeing enables people to cope with adversity, function effectively, have a sense of control and purpose over their life [36]. Psychosocial wellbeing is a broader construct that encompasses both psychological and social aspects of wellbeing. It includes factors such as social support, relationships, and quality of life [37]. Qualitative studies were mapped onto the domains by applying the MH and wellbeing outcome definitions to each qualitative study. The authors assessed which aspect of wellbeing or mental health outcome was described in the study findings. See Appendix D for tools used to assess mental health outcomes and the domain categories for included studies.

### Data extraction

**Fig. 1:**
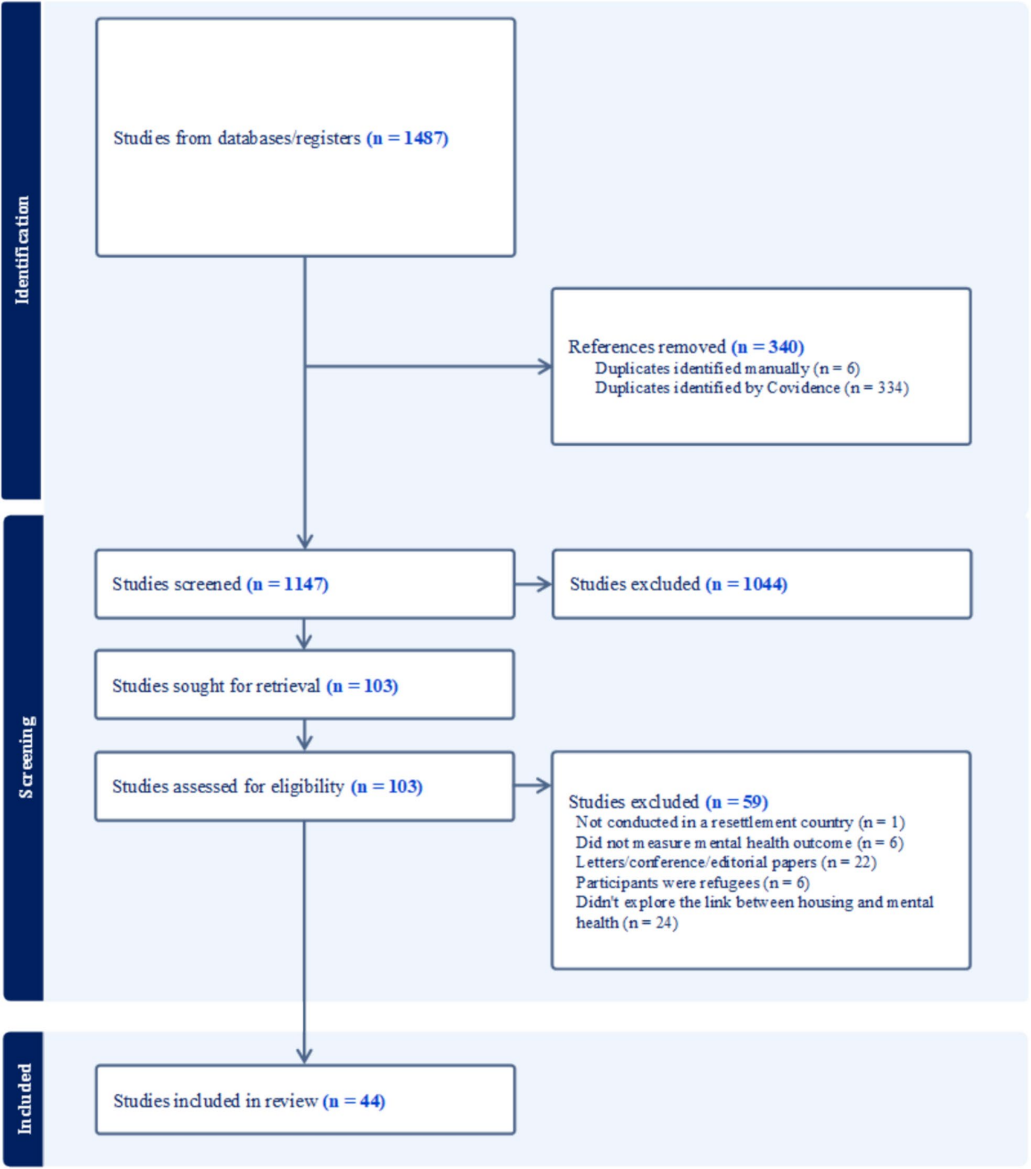
PRISMA Flow diagram of eligible studies

References were exported from EndNote to Covidence to manage study selection. Two independent researchers [SM and YL] screened studies in stages. First, 1,487 records were identified of which 340 duplicates were removed. After screening remaining titles and abstracts, 103 full text records were screened. References cited in systematic reviews were screened for relevant papers. Forty-four studies were included in the final selection. The PRISMA flow diagram [32] is shown in Fig. 1.

A data extraction form was constructed information was collected on methods, variable collection, measurement of exposure (housing affordability, security, suitability) and outcome (mental health and wellbeing) see Appendix B.

The first author synthesised the data thematically, using Braun & Clarke’s six-stage method [38]. First step was familiarisation with the data. Second, initial codes were generated manually with input from all authors. Third, further codes were developed iteratively, then collated into preliminary themes according to negative or protective effects on mental health. The relationships between codes were examined, compiled, and summarised for each theme. Fourth, initial themes across literature data were reviewed, splitting, combining, or discarding less meaningful ones as appropriate. Fifth, the authorship team defined and named final themes through discussion and further integration. Finally, we refined and contextualised themes during the reporting process.

## Results

Forty-four studies met the inclusion criteria. A description of the 44 studies is shown in Appendix D. Of these, most were conducted in Europe (n = 27). The remainder in Australia (n = 9), United States (n = 6) and Canada (n = 2). In terms of methodology, there were 22 quantitative studies, including 7 longitudinal. Additionally, there were 16 qualitative studies, 4 mixed methods studies. Two mixed methods papers published in grey literature. There were 20 studies where participants were exclusively refugees, ten were exclusively asylum seekers and fourteen were a mix where participants included refugees, asylum seekers and professionals or staff that worked with refugees. The length of time participants had been living in their new country varied across the studies. In 19 of the 44 studies, participants had lived in their new country for less than three years, in nine studies participants had been in the country between three and seven years, one study included a participant who had been in the country for 12 years. Of the remaining 15 studies, it was unclear how long people had been in the country. Most of the studies (86%) were conducted between 2012 and 2022.

We assessed the quality of included studies using the Joanna Briggs Institute (JBI) Critical Appraisal Checklist for Analytical Cross-Sectional Studies and Critical Appraisal Skills Program Qualitative Studies Checklist [39]. Qualitative studies rated well on the quality assessment however, only nine of 19 papers discussed ethics or indicated that they had ethics approval. Quantitative studies generally scored well against the assessment criteria of exposure, outcome, and statistical analysis, however only nine of 25 studies identified and accounted for confounders. Mental health measures in qualitative studies generally used self-description whereas quantitative studies used a range of standardised measurement tools, making direct comparison between these two types of studies difficult. See Appendix C for quality assessment.

## Housing and its impact on mental health and wellbeing

Overall, we found that policy was an overarching theme. The policy context of housing intersected with housing systems and settlement policies, establishing the conditions that facilitated or impeded accessibility, suitability, location and timing of housing for refugees and asylum seekers. Our thematic analysis of relevant articles identified four distinct dimensions of housing: Firstly, accessibility to safe and affordable housing. Secondly suitability of housing to meet the needs and expectations of refugees refers to the match between the housing provided and the needs and expectations of refugees, such as cultural considerations and family size. Thirdly, environmental aspects of housing which included quality and location of housing. Finally, we explored the timing of access to housing and the association with mental health and wellbeing.

The multiplicity and heterogeneity of mental health measures meant that specific housing factors were associated with a range of mental health and wellbeing outcomes. These included PTSD, depression, anxiety, life satisfaction, and participants’ descriptions of wellbeing. We grouped negative associations and described the outcomes in terms of negative or poor mental health. The following section of the paper will examine each of the identified housing dimensions and its associations with mental health and wellbeing looking at both risk and protective factors.

## Acessibility

The ability to access to safe, affordable and suitable housing for refugees and asylum seekers is dependent on the settlement and housing policy context in each settlement country. Our review identified several key factors influencing housing accessibility and its impact on mental health. In European countries, asylum seekers were provided housing upon reception through government owned/operated facilities such as disused army barracks, hostels, reception centres, and housing estates or accommodation sourced through the private sector. Thirty-five papers discussed the impact of government provided housing on refugee and asylum seeker mental health and found that government operated facilities were institutional-like and did not provide an environment that supported wellbeing or facilitated integration. The dispersal policy employed by the UK sent asylum seekers to housing estates in disadvantaged areas, with low quality, poorly maintained housing, and located in neighbourhoods where they felt unsafe, which was associated with negative mental health and wellbeing [40–50]. Additionally, in cases where there was a lack of permanent housing supply, refugees experienced extended stays in asylum reception facilities exacerbating poor mental health [49, 51, 52].

Whilst housed in government provided facilities, asylum seekers cited lack of control over where they lived, their roommates, their food, and the inability to cook their own culturally appropriate food as resulting in a sense of loss of autonomy and agency that negatively impacted their wellbeing [41, 44, 45, 53–64]. Policies at government reception centres, which imposed type and timing of leisure and recreation activities also reduced wellbeing [41, 53]. However, when allowed regular exercise or recreation, or access to a library or television, or to mix with the local community, asylum seekers reported improved wellbeing, and mental health [58, 62, 64, 65]. Many countries applied additonal policies restricting or preventing asylum seekers from working which increased financial hardship and negatively impacted their mental health [54, 55, 58, 66]

In the USA, Canada, and Australia, refugees and asylum seekers faced difficulties navigating private rental markets and experienced discrimination, negatively impacting their mental health. The participants groups of solely refugees or mixed refugee-asylum seekers and the use of ‘refugee’ as an umbrella term in these studies made it challenging to differentiate findings between the two populations. Poor mental health was associated with limited availability of affordable or suitable housing, neighbourhood violence, discrimination, and interpersonal conflict meant refugees were forced to move to find more suitable housing [17, 50]. Forced moves were associated with increased likihood of poor mental health and wellbeing [40, 41, 47, 52, 53, 66–68]. Those who did not need to move or were able to move to more desirable housing reported fewer mental health symptoms [40, 47, 60]. The lack of affordable housing was often cited as a reason for moving. Refugees faced challenges in securing and maintaining employment and government income support was deemed inadequate to cover rent, contributing significantly to financial and mental stress [14, 47, 69–71]. Involvement with employment and housing services, strong social support and community connections helped access housing which was found to improve refugee mental health and wellbeing [2, 57, 58, 65, 66, 71]. Additionally, a study in Canada that compared government and privately sponsonsored refugees found that privately sponsored refugees had better community support which was found to be a protective factor in refugee wellbeing [71].

## Suitability

The suitability of housing refers to how well a dwelling meets the needs and requirements of its occupants. A suitable home should provide adequate space, comfort, and functionality for its residents while also being affordable and in a safe environment [72]. Our analysis revealed several dimensions of housing suitability that significantly impact wellbeing. Fifteen of 44 studies identified that the lack of suitability of their housing to meet the specific needs (and expectations) of participants negatively impacted their mental health [42, 49, 51, 54, 59, 61, 62, 66, 68–70, 73–76]. Mental health and wellbeing were negatively impacted when refugees felt that they could not ask for what they considered necessary but instead felt they were told by service providers what they needed. They also reported feeling that housing providers were not responsive to or able to address their needs, and services lacked cultural awareness [42, 59, 75]. In some cases, after residency was granted to asylum seekers, instead of having stable housing they continued to experience unstable housing and forced moves, leading to disillusionment, frustration and poor mental health [49, 62, 68, 69]. When people attained better housing, they experienced a sense of satisfaction, safety and belonging which was associated with better mental health outcomes [61, 66, 73, 74, 76]. Overall, mental health suffered if housing did not meet people’s social, cultural, and family needs, including the expectation of stable housing [51, 54, 70].

The physical aspects of housing, including the quality of construction, design, and location can have significant impacts on occupants’ health [72]. Thirty-five of 44 papers investigated the physical conditions of refugee housing. Overcrowding and lack of privacy due to size of the house [17, 43, 49, 50, 52, 58, 66, 68, 77] or having to share rooms [41, 44, 51, 53–55, 57, 60–62, 64, 75] were most often found to negatively impact mental health. Studies focusing on asylum seekers were more likely to cite overcrowding as impacting mental health compared to those focusing on refugees [54, 62, 66, 75]. Houses that were in poor condition, unclean, required maintenance, and/or in a state of disrepair were also found to negatively impact mental health and wellbeing [14, 17, 40, 41, 45, 48, 50–52, 55, 62, 65, 69–71, 73, 74, 76]. Lack of heating and cooling was specifically associated with negative physical and psychological health [17].

## Location

The location of a property in relation to social connections, essential services and amenities is an essential in promoting good health. The concept of healthy housing is one that “supports social interactions, provides access to services and green spaces and facilitates the safe use of active and public transport options” [72]. Many refugees and asylum seekers were placed in disadvantaged neighbourhoods, which had higher crime rates, real or percieved lack of safety were associated with poorer mental health and wellbeing [14, 42, 43, 46, 47, 49, 51–55, 59, 60, 68, 70]. Fifteen studies cited location and safety of the neighbourhood as influencing wellbeing and ten identified suitability of amenities as an issue, for example lack healthcare or employment services, little or no access to public transport, no garden or outdoor space, or noisy location. Lack of open space and small living rooms meant that families had nowhere to exercise, play or socialise, which negatively affected both parents’ and children’s mental health and wellbeing [41, 46, 54, 55, 62, 77]. Lack of access to transport, schools, work, and healthcare facilities, and distance from a support network of community, family and friends contributed to feelings of isolation and disconnectedness, which negatively impacted mental health [44, 48, 52, 53, 64, 68].

In terms of protective factors, independent housing that was in good condition, clean, with a garden and adequate space to exercise contributed to better mental health and wellbeing [17, 55, 57, 60, 76]. A well-located neighbourhood that felt safe with access to public transport, healthcare and other services, had friendly neighbours and good social connections was also shown to improve life satisfaction and wellbeing [17, 55, 57, 58, 60, 62, 76, 77]. Asylum seekers living in reception centres in a decentralised location were better able to interact with the local community which strengthened integration and improved wellbeing [62].

## Time

The temporal aspects of housing, including the timing of access to housing support and the duration of stay emerged as an important dimension in refugee and asylum seeker mental health. Our analysis revealed several time-related factors that warrant consideration. We examined studies to see if they specifically addressed the relationship between housing factors and mental health of refugees and asylum seekers over time. Many studies focused housing very soon after arrival. For example, the negative impact on mental health and wellbeing when refugees faced an extended stay in asylum reception centres after being granted refugee status [51]. However, we did not find any studies that specifically examined housing factors and its association with mental health and wellbeing over time. Studies referencing time as a factor in mental health outcomes generally looked at number of moves and found that higher mobility was negatively associated with poor mental health [40, 46, 47, 52, 53, 66, 67]. A UK study found that 28 days given to find their own accommodation upon being granted their leave to remain was a crucial factor in risk of homelessness for refugees[45]. The combination of removal of government housing, lack of settlement support and integration policy meant that refugees had to begin the process of finding housing without support, 28 days was not enough time to complete this task [48, 55, 64, 68]. One study found refugees who had not moved during the preceding five years were less likely to have poor mental health [40]. Other studies found that time waiting for housing or protracted time involved in the refugee determination process or having a temporary visa negatively impacted mental health [46, 68]. Whereas being granted asylum or permanent residency was associated with improved mental health [65, 78]. A study in Australia found that unavailability of social housing led to waitlists of 16 years which was a trigger for poor mental health related to previous trauma of time spent waiting in refugee camps and feelings of rejection [69]. The limited research exploring housing factors and refugee settlement over time made it challenging to fully address the second research question. Specifically, we were unable to definitively determine if or when the timing of access to housing support offered the greatest benefits.

## Discussion

This review analysed research evidence describing how housing conditions are associated with refugee mental health and wellbeing in high-income resettlement countries. It found refugees experienced significant housing issues which contributed to poor mental health outcomes. This supports previous studies that identified housing as an important social determinant of health for refugees [10, 15]. Unsuitable housing conditions, including inadequate physical conditions and poor location, had a negative impact on mental health and wellbeing outcomes exacerbated by lack of autonomy. The lack of studies exploring housing factors across time in refugee settlement meant it was difficult to fully answer the second research question. We could not conclusively demonstrate if the timing of housing support or access provided any additional benefits. However, homelessness, which represents the most extreme experience of housing problems, was found to be a common occurrence among asylum seekers and refugees, particularly at time points when government assistance was ceased [17, 42, 45, 49, 52, 68, 69, 71, 76].

The challenges refugees face in accessing suitable housing reflect broader systemic issues in resettlement countries. Our findings suggest that housing policies often fail to account for the unique needs and vulnerabilities of refugee populations. Countries vary in the level of settlement support they provide to refugees for housing and other needs. Some offer comprehensive assistance, while others provide little to no help. Lack of settlement support can lead to difficulties in navigating housing systems and an inability to secure suitable housing. This can force refugees to move in search of affordable or appropriate accommodation. Forced moves, and dissatisfaction with housing can result in poor mental health. The inability to establish and maintain social connections, can create a sense of isolation and hinder integration, previous studies confirm this finding [79, 80].

In comparison, privately sponsored refugees in Canada, who received community-based support for housing, employment, and social integration, reported better mental health outcomes than their government-sponsored counterparts lacking equivalent assistance. These findings suggest that policy contexts significantly influence refugee integration into host societies, with long-term implications for both refugee populations and host communities. The association between housing instability and poor mental health outcomes highlights the critical need for more tailored housing support systems. This relationship may be bidirectional; poor mental health could also impede refugees’ ability to secure stable housing, creating a potential cycle of disadvantage.

Furthermore, we found that asylum seekers residing in reception centers, intentionally segregated from local communities, experienced an intensified sense of otherness and discrimination. This segregation was associated with diminished agency, lack of purpose, and limited social connections, collectively impeding integration and reducing psychological wellbeing. Previous research indicates that engagement in meaningful activities and the presence of supportive social connections can mitigate the negative emotional impacts of family separation and prolonged uncertainty [26]. Finding a job is considered one of the more meaningful activities. Allowing and supporting asylum seekers to seek employment would not only help with financial stability but also provide a sense of agency and control, improving mental health [81]. This underscores the need for a policy review to enable asylum seekers to work whilst awaiting the decision on their case. It also raises questions about the long-term impacts of forced destitution on asylum seeker mental health and wellbeing.

The importance of housing suitability extends beyond mere physical adequacy. Our findings indicate that culturally appropriate housing that meets family needs is crucial for mental wellbeing. This suggests that a one-size-fits-all approach to refugee housing is inadequate. The negative impact of unsuitable housing on mental health may be compounded by the stress of cultural adjustment, highlighting the need for housing solutions that facilitate, rather than hinder, the integration process. Supportive housing policies are central for successful integration of refugee populations [15, 82]. Service providers play an important role in creating a supportive environment for refugees. Refugees should be able to easily navigate services and access the settlement resources they need to thrive in their new communities. For example, to improve cultural competency housing services could be provided with training and resourcing for multilingual staff, interpreting and multilingual resources. Additionally, access to data systems that provide a more coordinated approach across settlement, housing and health sectors assists with information sharing decreasing the need for refugees to repeat their story helping reduce stressful experiences when engaging with services.

The significant impact of housing location on mental health and wellbeing underscores the importance of considering broader neighborhood characteristics in refugee resettlement. Our findings suggest that housing in areas with limited access to services, high crime rates, or lacking in community connections can exacerbate mental health issues. Previous research has shown that when refugees have connections to family and community, it promotes their sense of belonging and improves wellbeing. The ability to interact with the local community strengthens social connections providing additional social and emotional support as well as facilitating social cohesion and integration [79]. Suitable, stable, and well-located housing acts as a foundation for promoting wellbeing of refugee populations. This highlights the need for a more holistic approach to refugee resettlement that considers housing within the context of community resources and social networks.

The temporal aspects of housing support emerged as a critical yet often overlooked factor. Our findings suggest that the timing of housing interventions can significantly influence long-term mental health outcomes. Available evidence from a systematic review shows that prior exposure to housing disadvantage is consistently associated with poor mental health [83]. The duration of residency in the host country also plays a significant role in mental health outcomes. Refugees with longer stays often experience multiple relocations in search of more suitable accommodation, a pattern that can disrupt social connections and impede community integration. Studies exploring social networks found social connections to be highly beneficial in the search for housing, linking into information and opportunities within their community [84]. Asylum seekers experiencing protracted periods of uncertainty, characterised by unresolved asylum status, face significant impediments to future planning. This state of prolonged temporariness exerts a detrimental impact on their mental health and overall wellbeing[26, 85]. Our study confirmed previous findings showing that poor mental health was exacerbated if refugees had experiences of uncertainty or temporariness, like time spent in immigration detention, being on a temporary visa and living in a hostile political climate [14, 17, 42, 44, 46, 48, 49, 53, 55, 57, 58, 60, 64, 66, 75–77]. Refugees’ experiences can evolve over the course of their resettlement; this temporal aspect should be considered in resettlement policy.

Given that protracted periods of uncertainty are related to poor mental health and wellbeing the provision of stable housing could be one way to address this. Providing refugees and asylum seekers with a sense of security and safety serves as a foundation for rebuilding their lives and integrating into their new communities [22]. Successful integration, has a positive impact on mental health outcomes through access to employment, education, and income security, and opportunities to develop language skills thus building social connections [43–46, 57, 65, 67, 77]. These factors interact with housing and contribute to a sense of belonging and positive psychological wellbeing. This underscores the need for early, sustained housing support in the resettlement process. It also raises questions about the long-term impacts of initial housing experiences on refugee integration and wellbeing.

These findings collectively suggest that housing is not merely a physical determinant of health, but a complex social determinant that intersects with and influences other key factors such as employment and social support. The interrelated stressors of housing factors (access, suitability, and location), employment insecurity, and social support negatively impact refugees’ mental wellbeing and overall stability in host communities. Challenges in obtaining and retaining employment and housing instability reinforce “double precarity” [86], resulting in poor mental health. Inadequate income either from work or government support forces refugees to move further out creating further issues of access to jobs and services. The challenging process of securing accommodation, coupled with poor quality, overcrowded, and unhygienic housing conditions, that fails to meet refugees’ expectations of safe housing and improved living standards, leads to disappointment and frustration. The search for better quality accommodation resulting in constant moves disrupts social networks, increasing isolation and reducing wellbeing. Insecurity of tenure and lack of autonomy further contribute to poor mental health outcomes, as refugees feel disempowered and stressed about their living situations. The strong association between housing conditions and mental health outcomes implies that housing interventions could be a powerful tool for improving refugee mental health. However, the multifaceted nature of this relationship also suggests that effective interventions will require coordinated efforts across multiple sectors, including housing, health, and social services.

Moreover, our findings highlight a tension between current housing policies in many resettlement countries and the actual needs of refugee populations. Policies should consider both immediate and long-term housing needs to ensure comprehensive support throughout the resettlement process. With better coordination across housing and settlement sectors staged housing plans could be developed that evolve with refugees’ settlement journey, from initial accommodation to long-term, stable housing. The prevalence of housing issues across different national contexts suggests that this is a systemic issue requiring policy-level interventions. It also indicates that improving refugee housing conditions could have far-reaching benefits, not only for individual mental health but also for broader social integration and community cohesion.

A strength our study is the synthesis of evidence of housing domains related to mental health and wellbeing; it has provided insight into protective factors related to mental health. In strengthening our knowledge, it identifies opportunities for the application of future policy interventions. Much of the research focused on early post-arrival housing contexts and did not explore the effects on mental health and housing trajectories of longer-term, well-established refugees. There is a gap in our understanding of how housing impacts the mental health trajectories of refugees over the long term, and further research is needed to fully understand the relationship between the two. This is important because understanding the relationship over time can help develop targeted policy and interventions at crucial points, which could help protect refugee mental health, with significant implications for overall wellbeing and successful integration into society.

Our review has some limitations that should be considered in interpreting its findings. Our search was limited to English language papers, focused on refugees in specific settlement contexts rather than a broader category of forced migrants such as displaced people living within their own or a neighbouring country. Moreover, the academic literature may not provide an accurate reflection of the true landscape of practice in all settlement contexts. Due to the diversity of methodologies in the papers, we were not able to explore or draw conclusions about the impact of geographical or regional differences within or across countries. Further research into this would provide additional insights. A further limitation is in the definition of housing used—we adopted a wide definition, which meant the inclusion of studies with limited housing focus, which included those that only used a single-item measure relating to housing e.g. satisfaction with housing yes or no. Although asylum reception centres are a unique type of housing, and it is hard to compare them to other types of housing we included them so we could examine housing in different arrival contexts. As housing was our focus we did not include literature that explored homelessness, however we acknowledge that this is an extreme experience of lack of availability or affordability of housing. The inclusion of such studies may have provided further insights to mental health trajectories of refugees. Comparing mental health outcomes across varied study methods was difficult given the array of measurement tools and cultural backgrounds of the participants. The instruments used may not be culturally applicable or meaningful across all participant groups.

## Conclusions

Our scoping review reveals that numerous housing factors affecting the mental health outcomes of refugees and asylum seekers are modifiable through government or service provider interventions. The findings underscore the significant impact of housing on mental health, particularly highlighting known stressors during the early settlement period. Notably, the accessibility, suitability, and location of housing substantially contribute to poor mental health outcomes among refugees. For asylum seekers, the lack of autonomy and control over their environment is an additional factor contributing to poor mental health.

Given these insights, we recommend prioritising suitable housing in settlement policies. Additionally, asylum seekers should be granted more control and autonomy, including access to employment and greater choice over their living arrangements. This approach represents a promising path forward, potentially leading to improved mental health and well-being outcomes.

Key recommendations include:

1. Developing flexible housing policies that accommodate the diverse needs of refugees
2. Enhancing coordination and information sharing between housing, health, and settlement sectors
3. Providing more holistic approach to refugee resettlement that considers housing within the context of community resources and social networks
4. Provide early, sustained housing support in the resettlement process

Future research should focus on the long-term impacts of housing interventions on refugee mental health and integration outcomes. This knowledge will be crucial for developing targeted, effective policies that support refugee and asylum seeker wellbeing throughout the settlement process. By addressing these critical housing factors through evidence-based policies and targeted interventions, we can significantly improve the mental health outcomes and overall wellbeing of refugees and asylum seekers, thereby facilitating their successful integration and contributing to more inclusive, resilient, and cohesive communities in resettlement countries.

## Data Availability

The data supporting the findings of this scoping review are available in the supplementary materials, which include the full list of journal article titles reviewed. Additional data and materials are available from the corresponding author upon request.

## Appendix A

See Table 1

**Table 1:** Search terms

Medline Ovid	Refugee* OR asylum OR (humanitarian adj3 arrival*) OR (Humanitarian adj3 migra*) OR (forced adj3 migra*)	Housing OR accommodation OR (temporary adj3 accommodation) OR (housing adj3 program*) OR (housing adj3 assist*) OR (housing adj3 prov*) OR "housing intervention"OR (housing adj3 strategy) OR (housing adj3 policy) OR (social determinants adj2 health)	Mental health OR "mental ill*" OR mental disorder* OR (mental adj3 disorder*) OR (mental adj3 health) OR (mental adj3 ill*) OR "post traumatic stress disorder*" OR PTSD OR (anxiety OR anxious OR anx*) OR depress* OR (depress* adj3 disorder*) OR stress OR distress OR health OR (wellbeing OR wellbeing)
Web of science	TS = (refugee* OR asylum OR humanitarian NEAR/3 arrival* OR Humanitarian NEAR/3 migra* OR forced NEAR/3 migra*	TS = (housing OR accommodation OR temporary NEAR/3 accommodation OR housing NEAR/3 program* OR housing NEAR/3 assist* OR housing NEAR/3 prov* OR "housing intervention" OR housing NEAR/3 strategy OR housing NEAR/3 policy	TS = (mental health OR mental illness OR mental disorder OR mental NEAR/3 disORder* OR mental NEAR/3 health) OR mental NEAR/3 ill* OR post traumatic stress disORder* OR PTSD OR (anxiety OR anxious OR anx*) OR depress* OR depress* NEAT/3 disorder* OR stress OR distress OR health OR (wellbeing OR wellbeing)
PsychInfo	Refugee* or asylum or (Humanitarian adj3 migra*) or (humanitarian adj3 arrival*) or (forced adj3 migrant*)	Housing or accommodtion or (temporary adj3 accommodation) or (housing adj3 program*) or (housing adj3 assist*) OR (housing adj3 prov*) OR "housing intervention"OR (housing adj3 strategy) OR (housing adj3 policy) OR (social determinants adj2 health)	Mental health OR "mental ill*" OR mental disorder* OR (mental adj3 disorder*) OR (mental adj3 health) OR (mental adj3 ill*) OR "post traumatic stress disorder*" OR PTSD OR (anxiety OR anxious OR anx*) OR depress* OR (depress* adj3 disorder*) OR stress OR distress OR health OR (wellbeing OR wellbeing)
Embase	Refugee or asylum seeker or forced migrant	Housing	Health or mental health or wellbeing/ or anxiety/or anxiety disorder/ or mixed anxiety and depression"/ or depression/ or major depression/
SocINDEX	Refugees or asylum seekers or "humanitarian arrival"	Housing or home or accommodation or "housing intervention" or "housing policy" "housing provision"	Mental illness or mental health or mental disorder or wellbeing or wellbeing or well being

## Appendix B data extraction headings

### Study information

**Table:**

First Author	Year	Study Title	Country	Journal Name	Target audience	Funding	Study Design	Research question	Aims	Participant details	Recruitment Method

**Table:**

Sample size	Data Collection Method	Type of Housing/residence	Exposure Variable (housing) measurement tool	Housing factor	Length of time in host country	Duration of intervention/study	Focus of outcome (mental health/wellbeing)	Measurement tool	Analysis	Main findings	Implications	Limitations

### Synthesis of data extracted

Associations between housing factors and mental health and wellbeing

### Thematic categorisation of housing factors associated with mental health

**Table:**

Housing factor—negative assoc with MH	Housing factor—protective for MH	non-housing factors assoc with MH

### Quality of housing

**Table:**

Overcrowd/privacy/shared	Condition/maintenance/cleanliness	location/neighbourhood/safety	suitability/adequacy/noise/size/layout

### Access and systems

**Table:**

gov housing	legal system/asylum process, political climate	visa status/conditions	discrimination	affordability	Security (navigation/access issues/supply/forced moves/stability

### Individual needs and expectations

**Table:**

autonomy/agency/choice	expectations/attitude/perceptions	connections/relationships	language	existing MH	Self-esteem/insecurity/fear

## Appendix C

See Table 2

**Table 2:** Quality assessment of qualitative studies

First Author, Year	Aim	Methodology	Design	Recruitment	Data	Relationship	Ethics	Analysis	Findings	Value	Assessment
Smith, 2019	Yes	Yes	Yes	Yes	Yes	No	Can’t tell	No	Yes	Yes	7
Vitale, 2016	Yes	Yes	Yes	Yes	Yes	Yes	Yes	Yes	Yes	Yes	10
Due, 2019	Yes	Yes	Can’t tell	Yes	Yes	Yes	Can’t tell	Yes	Yes	Yes	8
Due, 2020	Yes	Yes	Yes	Yes	Yes	No	Yes	Yes	Yes	Yes	9
Gewalt, 2019	Yes	Yes	Yes	Yes	Yes	Yes	Yes	Yes	Yes	Yes	10
Gewalt, 2018	Yes	Yes	Yes	Yes	Yes	Yes	Yes	Yes	Yes	Yes	10
Hedstrom, 2021	Yes	Yes	Yes	Yes	Yes	Yes	Yes	Yes	Yes	Yes	10
Karlsson, 2019	Yes	Yes	Yes	Yes	Can’t tell	No	Can’t tell	Can’t tell	Yes	Yes	6
Miller, 2002	Yes	Yes	Can’t tell	Yes	Yes	Yes	Can’t tell	Yes	Yes	Yes	8
Nikolai, 2020	Yes	Yes	Can’t tell	Yes	Can’t tell	No	Yes	Can’t tell	Yes	Yes	6
Palmer, 2007	Can’t tell	Yes	Can’t tell	Yes	Can’t tell	Yes	Can’t tell	Can’t tell	Yes	Yes	5
Rowley, 2020	Yes	Yes	Can’t tell	Yes	Yes	No	Can’t tell	Yes	Yes	Yes	7
Silvus, 2016	Yes	Yes	Yes	Yes	Yes	No	Can’t tell	Can’t tell	Yes	Yes	7
Warfa, 2006	Yes	Yes	Yes	Yes	Yes	No	Can’t tell	Yes	Yes	Yes	8
Whitehouse, 2021	Yes	Can’t tell	Can’t tell	Yes	Can’t tell	No	Yes	Can’t tell	Yes	Yes	5
Ziersch, 2017	Yes	Yes	Yes	Yes	Yes	No	Yes	Yes	Yes	Yes	9
Murphy, 2020	Yes	Yes	Yes	Yes	Yes	No	Yes	No	Yes	Yes	8
Bernardes, 2011	Yes	Yes	Yes	Yes	Yes	No	Can’t tell	Yes	Yes	Yes	8
Hauge, 2017	Yes	Yes	Yes	Yes	Yes	No	Can’t tell	Can’t tell	Yes	Yes	7

## Appendix D

See Table 3

**Table 3:** Assessment of quantitative studies

First Author, Year	Inclusion criteria	Participants/setting	Exposure	Measurement	Confounders—identified	Confounders—accounted	Outcome	Statistical analysis	Assessment
Al Marsi 2021	Yes	Yes	Yes	Yes	No	No	Yes	Yes	6
Beza 2021	Yes	Yes	No	No	No	No	Yes	Yes	4
Bhui 2012	Yes	Yes	Yes	Yes	No	No	Yes	Yes	6
de Jong 2018	Yes	Yes	Yes	Yes	No	No	Yes	Yes	6
Eisen 2021	Yes	No	Yes	No	Yes	Yes	Yes	Yes	6
Haase 2019	Yes	Yes	Yes	Yes	No	No	Yes	Yes	6
Hynek 2020	Yes	Yes	Yes	No	No	No	Yes	No	4
Leiler 2019	Yes	Yes	Yes	Yes	No	No	Yes	Yes	6
Song 2015	No	Yes	Yes	Yes	No	No	Yes	Yes	5
Toscani 2005	Yes	Yes	Yes	Yes	No	Yes	Yes	Yes	7
Walther 2020	Yes	Yes	Yes	Yes	Yes	Yes	Yes	Yes	8
Walther 2020	Yes	Yes	Yes	Yes	Yes	Yes	Yes	Yes	8
Ahmad 2020	Yes	Yes	Yes	Yes	No	No	Yes	Yes	6
Ambrosetti 2021	Yes	Yes	Yes	Yes	Yes	Yes	Yes	Yes	8
Bakker 2016	Yes	Yes	Yes	Yes	Yes	Yes	Yes	Yes	8
Campbell 2018	No	Yes	Yes	Yes	Yes	Yes	Yes	Yes	7
Cooper 2019	Yes	Yes	Yes	Yes	No	No	Yes	Yes	6
Dudek 2022	Yes	Yes	Yes	Yes	Yes	Yes	Yes	Yes	8
Gillepsie 2020	Yes	Yes	Yes	Yes	No	No	Yes	Yes	6
Kivling-Boden 2001	Yes	Yes	Yes	Yes	No	No	Yes	Yes	6
Torlinska 2020	Yes	Yes	Yes	Yes	No	No	Yes	Yes	6
Whitsett 2017	No	Yes	Yes	Yes	No	No	Yes	Yes	5
Correa-Velez 2015	No	Yes	Yes	Yes	Yes	Yes	Yes	Yes	7

## Appendix E

See Table 4

**Table 4:** Descriptive table of included studies

First Author, Year	Country of origin, Host Country	Sample size (n)	Study Design	Housing Domain	Mental Health Domain	Assessment tools
Ahmad, 2020 [17]	Origin: Syria Host: Canada	baseline = 1924 year 2 = 1790	Quantitative-Longitudinal	Environment: housing conditions	Psychiatric disorder	Patient Health Questionnaire (PHQ-9); Multidimensional Scale of Perceived Social Support (MPSS)—structured interviews
Al Marsi, 2021 [54]	Origin: Syria Host: Germany	114	Quantitative—Cross sectional cohort	Policy: unsuitability government housing	Psychosocial and Psychological Wellbeing	WHOQOL-BREF Survey—interviews
Ambrosetti, 2021 [55]	Origin: Syria, Iraq, Afghanistan Eritrea Host: Germany	3957	Quantitative-Longitudinal	Environment: shared accommodation, privacy Policy: unsuitability government provided housing	Psychosocial wellbeing	Life satisfaction and SR-Health Surveys—interviews
Bakker, 2016 [43]	Origin: Afghan, Iraq, Iran, Somali, Host: Netherlands & UK	NL = 2975 UK = 921	Quantitative-Longitudinal and Cross-sectional	Policy: government housing Expectations: autonomy Environment: conditions, privacy	Psychosocial and Psychological wellbeing	Single question 5 point scale Mental Health = Very Good to Very Bad
Bernardes, 2011 [56]	Origin: Iran,Zimbabwe, Afghanistan, Iraq, Sri Lanka, Eritrea, Ethiopia Host: UK	29	Mixed methods	Policy: mobility Expectations: services understanding needs, discrimination Environment: shared accommodation, privacy	Psychosocial wellbeing Psychological wellbeing Psychological distress Psychiatric disorder	PMLD; GAD-7; CORE-OM; PSS-I Scales and free association narrative interviews
Beza, 2021 [57]	Origin: Syria Host: Greece	161	Quantitative—Cross sectional cohort	Policy: government housing Expectations: needs being met Environment: location of house	Psychological wellbeing	HRQoL—SF-36 Survey, MCS
Bhui, 2012 [38]	Origin: Somalia Host: UK	142	Quantitative—Cross sectional cohort	Policy: forced mobility Expectations: connection with relatives/friends, agency	Psychiatric disorder	Semi-structured interviews—MINI
Campbell, 2018 [68]	Origin: Afghanistan, DRC/Congo, Eritrea, Ethiopia, Iran, Iraq, Pakistan, Somalia, Sudan, Turkey Zimbabwe Host: UK	5678	Quantitative-Longitudinal	Expectations: satisfaction with housing, contact with relatives Environment: poor condition	Psychological wellbeing	HRQoL—SF-36 Survey—interviews
Cooper, 2019 [8]	Origin: Middle east, Southern Asia Host: Australia	W1 = 2,399 W2 = 2009 W3 = 1,894	Quantitative-Longitudinal	Policy: security, mobility Expectations: community	Psychiatric disorder	PTSD-8; HR-SMI: K6 Scales—telephone interviews
Correa-Velez, 2015 [64]	Origin: Middle east, Southern Asia Host: Australia	W1 = 120 W2 = 109 W3 = 100 W4 = 80 W5 = 51	Mixed methods	Policy: mobility Expectations: community support	Psychosocial wellbeing	Personal journal, self-described health and wellbeing; drawings and photos
de Jong, 2018 [76]	Origin: Iraq, Iran, Eritrea Host: US	278	Quantitative—Cross sectional cohort	Policy: security, mobility	Psychiatric disorder	CAFI-XC survey; HCL-25; PCL Scale – interview by mental health professional
Dudek, 2022 [58]	Origin: Syria, Afghanistan, Eritrea, Iraq Host: Germany	W1 = 574 W2 = 360 W3 = 411 W4 = 190	Quantitative-Longitudinal	Policy: unsuitable Expectations: agency/choice Environment: location, privacy	Psychological wellbeing	MCS—self described
Due, 2019 [44]	Origin: Iran, Afghanistan Host: Australia	8	Qualitative: PAR Photovoice	Policy: access, unstable housing Environment: safety	Psychosocial wellbeing Psychological wellbeing	Semi-structured interviews
Due, 2020 [17]	Origin: Africa, Middle East, Southeast Asia Host: Australia	11	Qualitative	Expectations: agency/choice, safety Environment: poor condition, suitability, location, noise, space	Psychological wellbeing	Photographs and interviews
Eisen, 2021 [77]	Origin: Ethiopia, Eritrea, Cameroon Host: US	78	Quantitative—Cross sectional cohort	Policy: unstable housing	Psychiatric disorder	PTSD-HTQ-30; HSCL-25; QOL surveys –interviews
Gewalt, 2019 [39]	Origin: West Africa, South Africa Host: Germany	9	qualitative	Policy: mobility Expectations: fear, agency/choice, insecurity Environment: shared accommodation, privacy cleanliness, noisy	Psychosocial wellbeing Psychological wellbeing Psychological distress	Semi-structure interviews
Gewalt, 2018 [51]	Origin: West Africa, South Africa Host: Germany	9	qualitative	Policy: government housing Expectations: self-determination/agency, sense of insecurity, Environment: shared accommodation (with other genders/cultures), privacy, location	Psychosocial wellbeing Psychological wellbeing	Semi-structure interviews
Gillepsie, 2020 [45]	Origin: Somalia Host: US	198	Quantitative -Longitudinal (over one year) cohort	Policy: Affordability, Stability, forced mobility, Expectations: interpersonal conflict, safety, security, discrimination Environment: location, unsafe neighbourhood, suitability (size)	Psychiatric disorder	HTQ Survey—interviews
Haase, 2019 [59]	Origin: Syria (+ 16 other) Host: Germany	94	Quantitative—Cross sectional cohort	Policy: government housing, discrimination Expectations: family, acculturation attitudes	Psychosocial wellbeing Psychological wellbeing	Acculturation Attitudes scale, 15-item scale from MIRIPS Questionnaire—interviews
Haigh, 2021 [69]	Origin: Syria, Iraq Host: Australia	not specified	Grey Literature—Impact assessment	Policy: government housing, discrimination access, mobility Expectations: family, attitudes, and expectations Environment: condition, suitability (size)	Psychosocial wellbeing Psychological wellbeing	Mental Wellbeing Impact Assessment Survey—interviews
Hauge, 2017 [60]	Origin: not stated Host: Norway	7	Mixed methods—case studies	Policy: government housing Expectations: expectations not being met Environment: condition, privacy, unsuitable	Psychosocial wellbeing Psychological wellbeing	Case studies
Hedstrom, 2021 [65]	Origin: Afghanistan, Iraq, Iran, Somalia, Kenya, Pakistan Host: Sweden	27	Qualitative—grounded theory	Policy: forced mobility Expectations: agency/autonomy, social contacts, sense of safety Environment: overcrowding, privacy	Psychosocial wellbeing Psychological wellbeing	Semi-structured interviews
Hynek, 2020 [74]	Origin: N/A Host: Austria	11	Quantitative fuzzy logic cognitive mapping	Policy: security, forced mobility Expectations: social contacts, political climate Environment: suitability (size), shared accommodation, privacy	Psychological wellbeing	Guided workshops
Karlsson, 2019 [52]	Origin: Not stated Host: Sweden	18	Qualitative Participatory rights-based approach	Policy: staff at government facility, Expectations: sense of safety, autonomy/choice Environment: overcrowding, privacy, safety	Psychosocial wellbeing Psychological wellbeing	Drawings/writing and informal conversations
Kivling-Boden, 2001 [70]	Origin: former Yugoslavia Host: Sweden	T1 = 35 T2 = 27	Quantitative longitudinal cohort	Expectations: satisfaction with housing Environment: satisfaction with housing	Psychiatric disorder	HTQ Questionnaire; The Life-In-Exile Questionnaire—interview by mental health professional
Leiler, 2019 [49]	Origin: Afghanistan, Iraq, Iran, Eritrea, Somalia Host: Sweden	510	Quantitative cross-sectional cohort	Policy: stability, availability, access Environment: conditions, location	Psychosocial wellbeing Psychological wellbeing Psychological distress Psychiatric disorder	PHQ-9; GAD-7; PC-PTSD; WHOQOL-BREF questionnaires—interviews
Miller, 2002 [71]	Origin: Bosnian Host: US	28	Qualitative	Policy: affordability, navigating Policy, Expectations: expectations, social and community loss Environment: overcrowding, privacy, adequate, condition, safety	Psychosocial wellbeing Psychological distress	The Refugee Distress and Coping Interview (RDCI)—narrative interview
Murphy, 2020 [46]	Origin: not stated Host: Northern Ireland	87	Mixed methods	Policy: forced mobility, navigating Policy, visa status, lack of work rights, service support Expectations: social isolation Environment: condition, inadequate/unsuitable	Psychosocial wellbeing Psychological wellbeing Psychological distress	Semi-structure interviews and focus groups
Nikolai, 2020 [72]	Origin: Syria Host: Switzerland	30	Qualitative cross-sectional	Expectations: sense of injustice Environment: overcrowding, privacy	Psychosocial wellbeing Psychological wellbeing Psychological distress	Free-listing, open-ended interviews
Palmer, 2007 [40]	Origin: Iraq, Somali, Iran, Ethiopia, Rwanda Host: UK	21	Qualitative	Policy: access (homelessness) Expectations: community, friends, family Environment: inadequate/unsuitable, isolation	Psychosocial wellbeing Psychological wellbeing	Semi-structured interviews
Rowley, 2020 [66]	Origin: Nigeria, Algeria, Albania, India, DRC, Cameroon Host: UK	9	Qualitative	Policy: forced mobility, instability, lack of support from government, navigating systems, lack of knowledge/resources Expectations: autonomy/choice, self-esteem, community (relationships) Environment: overcrowding, privacy, safety, location/neighbourhood	Psychosocial wellbeing Psychological wellbeing	Semi-structured interviews
Silvus, 2016 [67]	Origin: not stated Host: Canada	8	Qualitative -case study	Policy: affordability, mobility Expectations: community Environment: condition of house	Psychosocial wellbeing Psychological wellbeing	Interviews/focus groups and case study
Smith, 2019 [79]	Origin: Afghanistan, Bhutan, Burma, Sierra Leone, Sudan and Iran Host: Australia	24	Qualitative—PAR phenomenological approach	Policy: access	Psychological wellbeing	Focus groups and semi-structured interviews
Song, 2015 [78]	Origin: Iraq, Iran, Eritrea Host: US	278	Quantitative cross-sectional cohort	Policy: unstable Expectations: social isolation	Psychosocial wellbeing Psychological wellbeing Psychiatric disorder	PCL; HCL-25; GAF Scales—interviews
Torlinska, 2020 [41]	Origin: Middle east, Southern Asia Host: Australia	W1 = 1509 W2 = 1288 W3 = 1181 W4 = 1242	Quantitative—longitudinal	Policy: affordability Environment: condition, location	Psychological distress Psychiatric disorder	K6; PTSD-8 Scales—interviews
Toscani, 2005 [61]	Origin: Albania Host: Switzerland	580	Quantitative—cross-sectional	Policy: government housing, length of stay Expectations: family	Psychiatric disorder	MINI; SF-36; HTQ Surveys—interviews
Vitale, 2016 [47]	Origin: Iraq, Iran, Sudan, Eritrea, Morocco, Somalia Host: UK	9	Qualitative	Policy: access, forced moves, lack of gov support, lack of information/resources Expectations: powerlessness, sense of humiliation, Environment: safety, overcrowded, privacy, inadequate (size)	Psychosocial wellbeing Psychological wellbeing	Semi-structured interviews
Walther, 2020 [62]	Syria, Afghanistan, Iraq, Eritran, refugees and asylum seekers	4325	Quantitative cross-sectional cohort	Policy: government housing, length of stay, visa type, time in system Expectations: autonomy, community, language Environment: overcrowding, privacy	Psychosocial wellbeing Psychological wellbeing Psychological distress Psychiatric disorder	PHQ-4; Life Satisfaction Surveys—interviews
Walther, 2020 [42]	Origin: Syria, Afghanistan, Iraq, Eritrea Host: Germany	2569	Quantitative cross-sectional cohort	Policy: government housing, visa process Expectations: autonomy, community, language Environment: overcrowding, privacy	Psychiatric disorder	RHS-13;—Semi-structured interviews
Warfa, 2006 [50]	Origin: Somalia Host: UK	34	Qualitative	Policy: forced moves, mobility, prolonged stay in temp accommodation, access/availability, discrimination Expectations: agency/control, community, trust, disruption Environment: condition, location, overcrowded, privacy	Psychosocial wellbeing Psychological wellbeing	Discussion groups
Whitehouse, 2021 [53]	Origin: DRC, Cameroon, Afghanistan, Venezuela, Syria, Palestine, Iraq Host: Belgium	41	Qualitative	Policy: government housing Expectations: language, autonomy, meaninglessness, community sense of safety/security Environment: conditions, hygiene, community living, overcrowding, no personal space, privacy, location	Psychosocial wellbeing Psychological wellbeing	In-depth interviews
Whitsett, 2017 [48]	Origin: Iraq, Afghanistan, Ethiopia, Cameroon, DRC, Eritrea, Rwanda Host: US	105	Quantitative—longitudinal	Structural: unstable Environment: condition, overcrowded, privacy	Psychiatric disorder	HTQ; HSCL-25 Scales; HURIDOCS Events Standard Formats Tool – Semi-structured interviews
Ziersch, 2017 [73]	Origin: Afghanistan, Bhutan, Burundi, Iran, Sierra Leone, Sudan, Somalia Host: Australia	423	Qualitative	Policy: access/availability, affordability, mobility, insecurity (rental) Expectations: satisfaction, isolation, family Environment: condition	Psychosocial wellbeing Psychological wellbeing Psychological distress	SF-8 survey, interviews, and digital story telling
Ziersch, 2017 [63]	Origin: Middle east, Southern Asia Host: Australia	50	Qualitative	Policy: affordability, tenure (rental), mobility, discrimination, Expectations: sense of safety, agency/control, family Environment: condition, adequacy (size/layout), space, privacy,	Psychosocial wellbeing Psychological wellbeing	Semi-structured interviews

## Footnotes

1 The term refugee is used to describe people who have arrived in a resettlement country with, or been subsequently granted, humanitarian visas. Asylum seekers have left their home country and are awaiting the outcome of their application for protection in another country.
